# Robot-Assisted versus Laparoscopic Donor Nephrectomy: A Comparison of 250 Cases

**DOI:** 10.3390/jcm9061610

**Published:** 2020-05-26

**Authors:** Philip Zeuschner, Linda Hennig, Robert Peters, Matthias Saar, Johannes Linxweiler, Stefan Siemer, Ahmed Magheli, Jürgen Kramer, Lutz Liefeldt, Klemens Budde, Thorsten Schlomm, Michael Stöckle, Frank Friedersdorff

**Affiliations:** 1 Department of Urology and Pediatric Urology, Saarland University, Kirrberger Street 100, 66421 Homburg/Saar, Germany; philip.zeuschner@uks.eu (P.Z.); matthias.saar@uks.eu (M.S.); johannes.linxweiler@uks.eu (J.L.); stefan.siemer@uks.eu (S.S.); michael.stoeckle@uks.eu (M.S.); 2 Department of Urology, Charité-Universitätsmedizin Berlin, Corporate Member of Freie Universität Berlin, Humbold-Universität zu Berlin, and Berlin Institute of Health, Charitéplatz 1, 10117 Berlin, Germany; linda.hennig@charite.de (L.H.); robert.peters@charite.de (R.P.); thorsten.schlomm@charite.de (T.S.); 3 Department of Urology, Klinikum am Urban, 10967 Berlin, Germany; ahmed.magheli@vivantes.de (A.M.); juergen.kramer@vivantes.de (J.K.); 4 Department of Nephrology, Charité-Universitätsmedizin Berlin, Corporate Member of Freie Universität Berlin, Humbold-Universität zu Berlin, and Berlin Institute of Health, Charitéplatz 1, 10117 Berlin, Germany; lutz.liefeldt@charite.de (L.L.); klemens.budde@charite.de (K.B.)

**Keywords:** minimally-invasive donor nephrectomy, robot-assisted surgery, laparoscopic surgery, kidney transplantation, organ donation, living kidney donation

## Abstract

Living kidney donation is the best treatment for end-stage renal disease, however, the best surgical approach for minimally-invasive donor nephrectomy (DN) is still a matter of debate. This bi-centric study aimed to retrospectively compare perioperative outcomes and postoperative kidney function after 257 transperitoneal DNs including 52 robot-assisted (RDN) and 205 laparoscopic DNs (LDN). As primary outcomes, the intraoperative (operating time, warm ischemia time (WIT), major complications) and postoperative (length of stay, complications) results were compared. As secondary outcomes, postoperative kidney and graft function were analyzed including delayed graft function (DGF) rates, and the impact of the surgical approach was assessed. Overall, the type of minimally-invasive donor nephrectomy (RDN vs. LDN) did not affect primary outcomes, especially not operating time and WIT; and major complication and DGF rates were low in both groups. A history of smoking and preoperative kidney function, but not the surgical approach, were predictive for postoperative serum creatinine of the donor and recipient. To conclude, RDN and LDN have equivalent perioperative results in experienced centers. For this reason, not the surgical approach, but rather the graft- (preoperative kidney function) and patient-specific (history of smoking) aspects impacted postoperative kidney function.

## 1. Introduction

Living kidney donation is the ultimate treatment for end-stage renal disease (ESRD) [1]. Since the first successful living kidney donation in 1955 was carried out by Murray et al., many advances in surgical techniques and immunosuppressive therapy have led to substantial improvements in life expectancy and quality of life, not only for kidney recipients, but also for kidney donors [2]. In particular, minimally-invasive approaches for donor nephrectomy (DN) have increased the incidence of living kidney donation since the first laparoscopic DN (LDN) in 1995 and the first robot-assisted DN (RDN) in 2000 [3,4,5]. Unfortunately, higher donation rates have not been able to compensate for higher demand, which has led to at least 120,000 patients worldwide waiting for a kidney transplant today.

Many variations of minimally-invasive DN techniques have been described so far. Apart from hand-assisted methods as a bridge to open surgery, DN has also been performed in a retroperitoneoscopic (hand-assisted) manner [6,7]. In line with shorter flank incisions for open DN (“minimally invasive” open DN), Gill et al. conducted the first LDN via a LESS approach (laparoendoscopic single site surgery) in 2008 and inserted all trocars through the umbilicus [8,9]. Others have even tried to perform DN as a NOTES (natural orifice transluminal endoscopic surgery), and Pietrabissa et al. were the first to report a transvaginal extraction of the kidney after RDN in 2010 [10]. Today, some high-volume centers have performed more than 100 RDNs or LESS single-port RDNs, and employ specialized robotic single-site platforms [11,12]. However, the robotic approach still accounts for less than 5% of all minimally-invasive DNs, with increasing incidence compared to conventional transperitoneal LDN at more than 50% [13].

Irrespective of this magnitude of variations, minimally-invasive approaches for donor nephrectomy represent the standard of care, and are recommended as “the preferential technique”, according to the current guidelines for renal transplantation of the European Association of Urology (EAU) [14,15]. Multiple studies have shown that LDN is superior to open DN (ODN) in terms of hospital stay or postoperative pain, but the operating and warm ischemia time (WIT) are longer [16]. Importantly, LDN is not inferior in terms of complication rates, short- and long-term graft function. On the other hand, when comparing LDN with the robotic approach, RDN appears to have even less postoperative pain and less blood loss, but a longer WIT and operating time [17]. Nonetheless, analyses of cohorts with big sample sizes are still lacking, and the high variability of minimally-invasive DN renders it difficult to draw direct conclusions.

With this in mind, we conducted a retrospective bi-centric comparison of transperitoneal LDN with RDN and included more than 250 interventions. We aimed to compare perioperative outcomes as well as short- and mid-term kidney function of the donor and recipient up to four years after surgery. Alongside sub-analyses controlling for inherent learning, regression analyses to predict postoperative kidney and graft function were performed. All LDNs were conducted at the largest German kidney transplant program run by a urologic department that has been performing LDNs since 1999. All RDNs including the very first RDN in Germany in 2007, were performed at a urologic department highly specialized in robotic surgery [18].

## 2. Materials and Methods

In total, 257 DNs performed at two tertiary referral centers were retrospectively analyzed. All 205 LDNs were conducted by 11 surgeons with a median caseload of 11 (range 2–43) at a urologic department specialized in laparoscopic kidney surgery including LDNs. The 52 RDNs were performed at another urologic department, which is specialized in robotic surgery in general. All RDNs were conducted by five surgeons with a median caseload of 10 (range 2–29). The interventions were performed in a transperitoneal fashion between 2007–2020 (RDN) and 2011–2016 (LDN).

At the robotic department, the very first RDN in Germany was conducted [18]. Before 2007, all donor nephrectomies had been held in an open fashion, so none of the robotic surgeons had prior expertise in LDN, but in a large variety of other robotic interventions. Thereafter, DN was standardized to a robot-assisted approach. The other department in this study has been performing LDNs since 1999. Both departments always conducted DNs in a minimally-invasive fashion during the study period, unless the donor had a significant amount of prior abdominal surgeries and consequently high risk for conversion. The corresponding kidney transplantations were held in an open fashion, except for the last 18 (34.6%) cases at the robotic department. As a part of the EAU-RAKT working group (European Association of Urology working group for robotic kidney transplantation), the first RAKT in Germany was performed there in June 2016 [19,20]. From then, all RDNs were followed by RAKTs.

This entire analysis was conducted in adherence with the correct scientific research work terms of the Charité Medical University of Berlin and Saarland University including full anonymization of patient data. All the patients included in the analysis provided written informed consent.

### 2.1. Surgical Technique

All RDNs were performed using a transperitoneal approach, with either a DaVinci^®^ Si or X system with four arms. The ports were placed pararectally. For the first RDNs, the graft was removed in a hand-assisted manner without a specimen bag via a Pfannenstiel incision, and later on via a periumbilically placed GelPOINT^®^ trocar (Applied Medical, Los Angeles, CA, USA). For LDN, the approach was purely laparoscopic, without the hand-assisted technique, which has been described previously [21,22]. In brief, four ports were used, and the kidney was extracted through an enlarged lateral trocar incision measuring 5 to 6 cm.

### 2.2. Data Collection and Outcome Measures

For the donor characteristics, age, gender, body mass index (BMI, kg/m²), pre-existing arterial hypertension, diabetes, and history of smoking were obtained. The graft’s side, scintigraphic split-renal function (DTPA), and number of arteries and veins served as organ-specific factors. For the recipient characteristics, age, gender, BMI, implantation side, and individual number of prior kidney transplantations were obtained.

Intraoperative (operating time, WIT, complications) and postoperative (length of stay, major postoperative complications based on Clavien–Dindo grade ≥3 within 30 days after surgery) results were analyzed as *primary outcomes*. The comparison and prediction of postoperative kidney function of the donor and of the recipient up to four years after transplantation served as *secondary outcomes*. Delayed graft function (DGF), defined as dialysis within one week after transplantation or insufficient serum creatinine decline not below 2 mg/dL, was analyzed as a further kidney-related secondary outcome.

### 2.3. Statistical Analysis

Primary and secondary outcomes were compared between the LDN and RDN group. To assess whether perioperative outcome was affected by an inherent learning curve, both groups were split in half and the outcomes were compared within each group. The first 34 (65.4%) RDNs were followed by an open transplantation, but the last 18 (34.6%) were followed by a robot-assisted kidney transplantation. To ensure that RAKT did not affect the perioperative results of RDN, the last 18 RDNs were excluded in another sub-analysis. The impact of patient-, graft- or surgery-specific factors on postoperative kidney function of the donor at discharge was assessed by linear regression analysis. To predict kidney function of the recipient one week after surgery, donor and recipient characteristics, DN, and transplantation-specific aspects were included in another uni- and multivariate regression analysis.

Categorical variables were reported as frequencies and proportions, and continuous data as the median and range. Fisher’s exact test and the Mann–Whitney U test were used to compare between groups. Covariates were included in the multiple regression analysis only if their respective effect was significant in the univariate analysis. The statistical analysis was performed by SPSS version 25 with Fix pack 2 installed (IBM, Armonk, NY, USA). All tests were two-sided, and *p*-values < 0.05 were considered significant.

## 3. Results

### 3.1. Overall Results: Primary Outcomes

In the RDN and LDN groups, most kidney donors were female (63–68%), 51–54 years old, and had a BMI of 25.4–25.9 (see Table 1). Donor characteristics only differed concerning the individual history of smoking, as there were more smokers in the LDN group (52.7 vs. 9.6%, *p* < 0.001). Donor organs were 20% right-sided and had a split-renal function of 50%. The number of organs with multiple arteries was no different between RDN and LDN (11.5% vs. 18.5%), but significantly more grafts in the LDN group had multiple veins (12.7% vs. none, *p* < 0.01). The groups did not differ regarding recipient characteristics. Most were male (67–70%), 42–45 years old, and had a BMI of 24.7–25.3. For more than 90% of recipients, it was their first kidney transplantation.

Concerning primary outcomes, neither the median operating time (RDN 223.5 vs. LDN 213 min), WIT (3 vs. 2.45 min), nor intraoperative complication rate (5.7 vs. 2.9%) were significantly different between groups (see Table 2). One RDN had to be converted to open surgery because of massive obesity and multiple trocar dislocations. In two other cases, a malfunction of the stapler and a lumbal vein caused bleeding, which could be managed robotically without the need for blood transfusions. In the LDN group, in one case, bleeding from a dorsal branch of the renal vein could not be controlled laparoscopically, leading to a conversion to open surgery. In another LDN case, the renal vein was torn during kidney removal, but could be reconstructed. Once, the donor’s spleen and the renal parenchyma were accidentally cut, and a small hole in the descending colon had to be sutured. A previously undetected obstructed ureteropelvic junction made one pyelovesicostomy necessary for a recipient in the LDN group.

The median length of stay of five days was no different between the LDN and RDN groups, nor was the postoperative major complication rate. In the RDN group, one patient had an ileus that dissolved after gastroscopy. In the LDN group, a bronchoscopy had to be performed because of dyspnea, and a retention of chylous ascites had to be punctured. In another case, continuous arterial bleeding from the abdominal internal oblique muscle made electrocoagulation necessary in the LDN group.

### 3.2. Learning Curve

When comparing the first half of the RDNs with the second half to analyze for inherent learning effects, the WIT, intra- and postoperative complication rate, and length of stay remained unchanged (see Table 3). Operating time significantly increased from 185 to 265 min in the RDN group (*p* < 0.001). This difference no longer remained significant when the last 18 RDN cases were excluded; in these cases, RDN was followed by robot-assisted kidney transplantation (185 vs. 226 min, n.s.). In the LDN group, the surgical results remained unchanged over time.

### 3.3. Kidney Function of the Donor and Recipient: Secondary Outcomes

**Figure 1:**
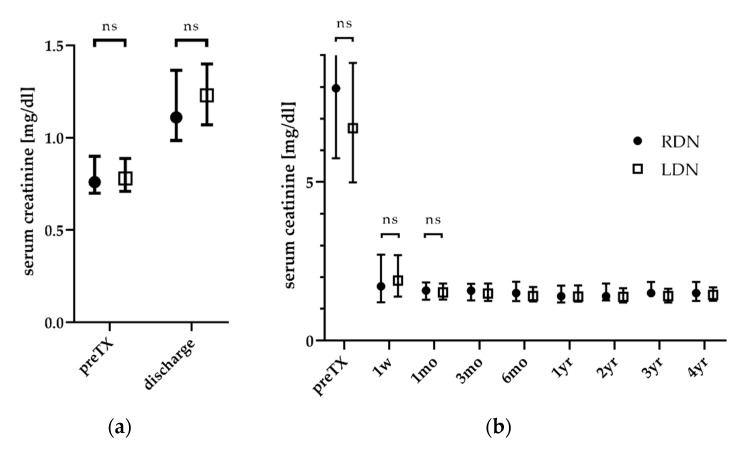
Follow-up of kidney function of the donor (**a**) and graft (**b**). The kidney function did not differ between robot-assisted (RDN) and laparoscopic donor nephrectomy (LDN).

The type of surgical approach of DN did not impact the postoperative kidney function either of the donor or the recipient (see Figure 1). Among the donors, kidney function did not differ preoperatively or at discharge between groups. For recipients, kidney function significantly improved after transplantation, irrespective of the type of DN, and stayed stable thereafter.

DGF rates were 6.3 to 11.5% (LDN vs. RDN), and did not significantly differ between groups and did not change over time (see Table 2 and Table 3). In the RDN group, DGF was caused by three (5.7%) suspected transplant renal artery stenoses, one (1.9%) perirenal hematoma due to double anticoagulation of the mechanic aortic valve and prolonged serum creatinine decline (no dialysis needed), one (1.9%) prolonged CIT (cold ischemia time) due to vascular complications during transplantation, and one (1.9%) insufficient serum creatinine decline without other cause. In the LDN group, DGF resulted from seven (3.4%) acute rejections, one (0.5%) lesion of the arterial anastomosis after the Fogarty maneuver, and one (0.5%) case of donor-related pre-existing vascular damage. One (0.5%) patient needed dialysis for depletion of potassium only, and in three (1.5%) other cases, the cause for DGF in the LDN group was unknown.

In the multivariate regression analysis, only patient-specific factors were found to have an impact on postoperative kidney function, but not surgical factors (see Table 4). Concerning the kidney function of the donor at discharge, male patient gender was predictive for worse kidney function (*B*-value 0.14, *p* < 0.001). Furthermore, worse preoperative kidney function was associated with worse postoperative function (*B*-value 1.0, *p* < 0.001). A history of smoking only had an impact on postoperative kidney function in the univariate analysis. No other (surgical) factors such as approach (LDN vs. RDN), operating time, intraoperative complications, WIT, kidney side, or number of arteries or veins, had an impact on the kidney function of the donor at discharge.

A history of donor smoking also had a significant impact on the kidney function of the recipient in the multivariate regression analysis: a kidney donor with a history of smoking caused worse graft function one week after transplantation (*B*-value 0.63, *p* < 0.05, see Table 4). Again, the preoperative kidney function of the recipient was predictive for their postoperative graft function (*B*-value 0.22, *p* < 0.001). In the univariate, but not the multivariate analysis, a preemptive kidney transplantation predicted better graft function (*B*-value −0.72, *p* < 0.05). Again, no surgical factors, either the type of donor nephrectomy (LDN vs. RDN) or the type of transplantation (open vs. robot-assisted), had an impact on graft function one week after transplantation.

## 4. Discussion

In this bi-centric study, a comparison of 257 minimally-invasive donor nephrectomies with 205 laparoscopic and 52 robot-assisted DNs was conducted. Of note, this analysis included the very first RDN in Germany, and all LDNs were performed at a urologic department where LDNs have been conducted since 1999 [18].

Concerning the primary outcomes, operating time was no different between RDN and LDN (223.5 vs. 213 min, see Table 1). Most studies describe shorter operating times for LDNs, but report highly variable results [17]. Mean operating times for RDNs range from 144 to 306 min [23,24], and for LDNs between 178 and 270 min [25,26], even when only studies with cohorts larger than 100 patients are included. These differences could result from inherent learning curves: Horgan et al. and Janki et al. have shown that operating times in RDN shorten with growing expertise [27,28]. Interestingly, our data do not show an inherent learning effect, either in the RDN or in the LDN cohort. Outcomes remained unchanged over time (see Table 3). Conversely, operating time became significantly longer within the second half of the RDNs (185 vs. 265 min, *p* < 0.001).

This counterintuitive development resulted from the way transplantations were organized, as both institutions perform DNs and transplantations in different operating rooms simultaneously, but not sequentially. Two surgical teams work in parallel, but the graft is not removed unless the transplantation team is ready, to avoid long cold ischemia times. The RDN cohort not only comprised the first RDN, but also the first robot-assisted kidney transplantation in Germany (procedure #35) [18,20]. Operating times in the RDN cohort became longer from that point, as the learning curve for RAKTs had not yet been passed. Naturally, the RDN team started more than 30 min before the transplantation team, but RAKT proved to be much more challenging and time-consuming. When excluding the last 18 cases, when RDN was followed by RAKT, the operating times of the RDNs did not change over time. Thus, the obvious lack of a typical learning curve illustrates that for LDNs, the learning curve had already been passed and for RDNs, significant prior expertise in robotic surgery made it possible to reach stable results from the start [29].

As with the operating time, WIT was not different between RDNs and LDNs (3 vs. 2.45 min). In the RDNs, most grafts were extracted via a GelPOINT^®^ trocar (Applied Medical, Los Angeles, CA, USA), which is an easy and fast, yet expensive method. Wang et al. illustrated significantly longer WIT for RDNs than LDNs in their meta-analysis, which is an often-stated argument against RDNs [17,30]. However, it is unlikely that differences of 30 or 60 s in WIT will harm the graft function in the long-, mid- or even short-term. It has clearly been shown that a WIT longer than 45 min impairs graft survival in living kidney donation [31]. Fortunately, neither our results nor those from other studies have documented WIT longer than 15 min for RDNs, keeping in mind that the consecutive CIT is again followed by another WIT during transplantation.

Intraoperative complication rates were low in both RDNs (5.7%) and LDNs (2.9%), and did not significantly differ. In line with others, most intraoperative complications were bleedings, whereof one in the LDN group made a conversion to open surgery necessary, but none in the RDN group [17]. In contrast, a patient with massive obesity had multiple trocar dislocations within the first minutes of surgery, so the RDN had to be converted to open surgery. Due to a technical defect of the stapler system for one patient in the RDN group, which made it cut but not staple, locking Hem-o-Lok clips were predominantly used later on, as described elsewhere [32]. During LDNs, Hem-o-Lok and titanium clips are used for the renal artery, a stapler for the right vein, and two Hem-o-Lok clips for the left vein. Not only intraoperative but also postoperative complication rates, according to Clavien–Dindo, were low and did not differ between LDN and RDN. Therefore, both surgical approaches had equivalent complication rates, while LDN has less costs, but RDN appears to be superior in complex situations such as bleedings.

The kidney donors were discharged five days after DN, irrespective of the type of surgery (see Table 2). Consequently, the median length of stay was longer than in most other works, ranging from 2–3 days for LDNs and RDNs [11,17,24]. This can be attributed to differences in national health care systems as (i) the German reimbursement system covers a longer hospital stay and (ii) most donors wanted to stay longer as inpatients for psychological reasons. In fact, only 15 (5.8%) patients were discharged two or three days after DN. Early discharge after RDN and LDN is possible from a surgical point of view, however, it has not been a crucial parameter for our perioperative approach, as long as neither patient satisfaction nor health care costs are affected.

As a secondary outcome, the impact of the surgical approach on postoperative kidney function was assessed. Kidney donors had a worse kidney function at discharge, which was comparable between groups and similar to results found in other studies (RDN 1.1 mg/dL vs. LDN 1.23 mg/dL; see Figure 1) [28,33]. Correspondingly, the preoperative kidney function, but not the type of surgical approach for DN, was predictive for the postoperative kidney function of the donor at discharge (see Table 4). Interestingly, patient gender also had a significant impact on postoperative kidney function. However, this should not be over-interpreted, as male kidney donors had a worse kidney function than women, with higher serum creatinine values preoperatively (0.9 vs. 0.72 mg/dL, *p* < 0.001) and postoperatively (1.42 vs. 1.1 mg/dL, *p* < 0.001) in this analysis. For this reason, (male) patient gender was predictive for (worse) postoperative kidney function; this may not be representative for other cohorts.

Similarly, Benoit et al. created a model to predict 1-year postoperative renal function of kidney donors after LDN, which has been externally validated [34,35]. The authors predicted postoperative eGFR by preoperative eGFR and patient age (postoperative eGFR = 31.71 + (0.5 × preoperative eGFR) − 0.314 × age at donation). In our model, patient age was not predictive for postoperative kidney function, potentially because we evaluated the short-term kidney function at discharge and not one year after DN.

Concerning recipients, the DGF rates of 6.3% (LDN) and 11.5% (RDN) did not significantly differ between groups. In general, there is a large variety of reported DGF rates in living kidney donation, ranging from 4 to 10% [36,37]. This not only results from center-specific differences, but also from inconsistent definitions: DGF can be defined by urine output per day, serum creatinine decline, or the need for dialysis after transplantation [36]. We applied a considerably broad definition for DGF (postoperative dialysis within one week after transplantation for any cause or insufficient creatinine decrease not below 2 mg/dL). DGF rates in the RDN group were 11.5% due to transplantation-related surgical, mainly vascular causes. One (1.9%) patient with a mechanic aortic valve developed a perirenal hematoma, causing prolonged creatinine decline without the need for dialysis. In the LDN group, DGF was mainly caused by acute rejections (3.4%), and also comprised one patient (0.5%) who required dialysis for potassium depletion only. Consequently, DGF did not result from the type of DN, but rather transplantation-specific causes.

Regardless, the kidney function of the recipients significantly improved after transplantation, and did not differ between groups during follow-up (see Figure 1). In the multiple regression analysis, not only the preoperative kidney function of the recipient, but also a history of donor smoking, had a significant impact on graft function one week after transplantation (see Table 4). Smoking is a well-known modifiable risk factor for the development of chronic and end-stage kidney disease [38,39]. A history of donor smoking has a negative impact not only on the survival of the donor, but also of the recipient [40]. In our cohort, a positive history of donor smoking increased serum creatinine one week after transplantation by 0.63 mg/dL. This highlights, again, the importance of informing not only transplant patients, but also potential kidney donors, about the risks of tobacco use, and the importance of helping patients to stop smoking.

This analysis is not devoid of limitations. As a bi-centric study, experienced but different surgeons and different teams conducted the RDNs and LDNs. Patient cohorts did not significantly differ in terms of characteristics, but were not equally balanced in terms of caseload. Although surgical results were not affected by inherent learning curves, at least the results in the RDN group were affected by simultaneous robot-assisted kidney transplantation. This procedural aspect highlights the complexity of comparing minimally-invasive donor nephrectomies: the surgical part itself is in high demand, but the high variability of the technical, procedural, and underlying ethical aspects also have to be taken into account [41].

## 5. Conclusions

Minimally-invasive surgical techniques have increased the acceptance of living kidney donation, but its high variability renders head-to-head comparisons of surgical approaches a complex task. In this bi-centric study, we compared more than 250 cases of 52 transperitoneal robotic DNs with 205 laparoscopic DNs. Operating time and length of stay were no different between groups, but slightly longer than elsewhere, as DNs and transplantations were conducted simultaneously to reduce CIT, and most other national health systems do not allow longer inpatient stays. Other perioperative results (complication rates, WIT) and mid-term kidney function including DGF rates were comparable with published data, and did not differ between RDN and LDN. This was possible because both centers already had prior expertise in either LDN itself or robotic surgery in general. For this reason, patient-specific factors (preoperative kidney function, history of donor smoking) were the more relevant impacts upon donor and graft function.

## Figures and Tables

**Table 1:** Comparison of donor, graft, and recipient characteristics.

	RDN (*n* = 52)	LDN (*n* = 205)	*p*-Value
**donor**			
age (yr)	54 (20; 70)	51 (21; 78)	n.s.
male gender	16 (30.8%)	75 (36.6%)	n.s.
BMI (kg/m²)	25.4 (17.6; 36.7)	25.9 (17.6; 36.1)	n.s.
pre-existing			
hypertension	15 (28.8%)	44 (21.5%)	n.s.
diabetes	1 (1.9%)	3 (1.5%)	n.s.
history of smoking	5 (9.6%)	108 (52.7%)	<0.001
**graft**			
right side	11 (21.2%)	45 (22%)	n.s.
multiple arteries	6 (11.5%)	38 (18.5%)	n.s.
multiple veins	0	26 (12.7%)	<0.01
scintigraphic function	50% (39; 57)	50% (38; 58)	n.s.
**recipient**			
age (yr)	42 (18; 66)	45 (6; 76)	n.s.
male gender	35 (67.3%)	144 (70.2%)	n.s.
BMI (kg/m²)	25.1 (17.6; 37)	24.7 (16.8; 40.8)	n.s.
side left	8 (15.4%)	46 (22.4%)	n.s.
first transplantation	48 (92.3%)	187 (91.2%)	n.s.

**Table 2:** Outcomes of 257 donor nephrectomies.

	RDN (*n* = 52)	LDN (*n* = 205)	*p*-Value
**Intraoperative**			
operating time (min)	223.5 (127; 363)	213 (120; 392)	n.s.
WIT (min)	3 (0.5; 1)	2.45 (0.4; 5.27)	n.s.
complications	3 (5.7%)	6 (2.9%)	n.s.
conversions	1 (1.9%)	1 (0.5%)	n.s.
**postoperative**			
length of stay (d)	5 (2; 12)	5 (3; 18)	n.s.
Clavien–Dindo			n.s.
grade 3	1 (1.9%)	1 (0.5%)	n.s.
grade 4	-	2 (1%)	n.s.
grade 5	-	-	n.s.
**recipient**			
DGF	6 (11.5%)	13 (6.3%)	n.s.

**Table 3:** Assessment for the inherent learning curves in RDN and LDN by comparing the first with the second half of cases within each group.

		RDN			LDN	
	1^st^ half (*n* = 26)	2^nd^ half (*n* = 26)	*p*	1^st^ half (*n* = 102)	2^nd^ half (*n* = 103)	*p*
**Intraoperative**						
operating time	185 (148; 284)	265 (127; 363)	<0.001 ^1^	213 (135; 392)	216 (120; 363)	n.s.
WIT (min)	3 (0.5; 9)	2 (1; 10)	n.s.	2.4 (0.4; 5)	2.5 (0.5; 5.2)	n.s.
complications	2 (7.7%)	1 (3.8%)	n.s.	3 (2.9%)	3 (2.9%)	n.s.
conversions	1 (3.8%)	-	n.s.	-	1 (0.9%)	n.s.
**postoperative**						
length of stay (d)	5 (3–12)	5 (2–7)	n.s.	5 (3; 18)	5 (3; 11)	n.s.
Clavien–Dindo	0 (0; 2)	0 (0)	n.s.	0 (0; 4)	0 (0; 4)	n.s.
grade 3	1 (3.8%)	-	n.s.	1 (1%)	-	n.s.
grade 4	-	-	n.s.	1 (1%)	1 (1%)	n.s.
grade 5	-	-	n.s.	-	-	n.s.
**recipient**						
DGF	4 (15.4%)	2 (7.7%)	n.s.	6 (5.9%)	7 (6.8%)	n.s.

^1^ When excluding the last 18 cases, where RDN was followed by robot-assisted kidney transplantation, the difference was no longer significant (185 vs. 226 min, n.s.).

**Table 4:** Multivariable regression analysis to predict the serum creatinine (1) of the donor at discharge (“donor kidney function”) or (2) of the recipient one week after transplantation (“graft function”).

Variable	*B*-Value	*p*-Value
**donor kidney function**		
gender	0.14 (0.09; 0.19)	<0.001
preTX serum creatinine	1.00 (0.82; 1.18)	<0.001
surgical approach	-	n.s.
**graft function**		
smoking donor	0.63 (1.21; 0.05)	<0.05
preemptive Tx	-	n.s.
preTX serum creatinine	0.22 (0.12; 0.31)	<0.001
surgical approach	-	n.s.
